# Next-generation biotechnology inspired by extremes: The potential of extremophile organisms for synthetic biology and for more efficient and sustainable biotechnology

**DOI:** 10.1038/s44319-025-00389-6

**Published:** 2025-02-27

**Authors:** Shuang Zheng, Mingwei Shao, Wanze Wang, Guo-Qiang Chen

**Affiliations:** 1 School of Life Sciences, Tsinghua University, 100084 Beijing, China; 2 Key Lab of Industrial Biocatalysts of the Ministry of Education, Department of Chemical Engineering, Tsinghua University, 100084 Beijing, China; 3 Tsinghua-Peking Center for Life Sciences, Tsinghua University, 100084 Beijing, China; 4 Center for Synthetic and Systems Biology, Tsinghua University, 100084 Beijing, China

**Keywords:** Biotechnology & Synthetic Biology, Economics, Law & Politics, Microbiology, Virology & Host Pathogen Interaction

## Abstract

Research on and exploitation of the genetic diversity of bacteria adapted to thrive under extreme conditions holds great potential for biotechnology to develop more efficient, cheaper and more sustainable production processes.

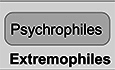

Extremophiles describe a wide range of microorganisms that are able to survive and grow under extreme environmental conditions with physical or chemical parameters that are way beyond the typical ‘comfort zone’ for most organisms (Shu and Huang, 2022). Their abilities to withstand extreme temperatures, pH values or salt concentrations make extremophiles unique in terms of morphology, structure, physiology, biochemistry, and evolutionary mechanisms; the structures of proteins, nucleic acids, lipids, and the characteristics of enzymes differ from microorganisms living under normal conditions (Shu and Huang, 2022). Research on extremophiles has not only given fascinating insights into the origin and evolution of life but it also facilitates what is termed ‘Next-Generation Industrial Biotechnology’ that aims to develop more efficient, energy-saving production processes based on cheap and easily available substrates (Shu and Huang, 2022; Wang et al, 2024).

Research on extremophiles […] facilitates what is termed ‘Next-Generation Industrial Biotechnology’ that aims to develop more efficient, energy-saving production processes …

With advances in DNA sequencing, genetic engineering technology, synthetic biology tools are becoming increasingly accessible and adaptable for use in non-model organisms (Ye et al, 2023; Blombach et al, 2022). By leveraging the unique genetic backgrounds and metabolic pathways of extremophiles, synthetic biologists can design biosynthetic pathways to obtain bioproducts, thereby achieving economic and societal benefits, as observed with the rise of biotech companies based on *Halomonas* (Ye et al, 2023).

## Extremophiles and resource mining

**Figure 1:**
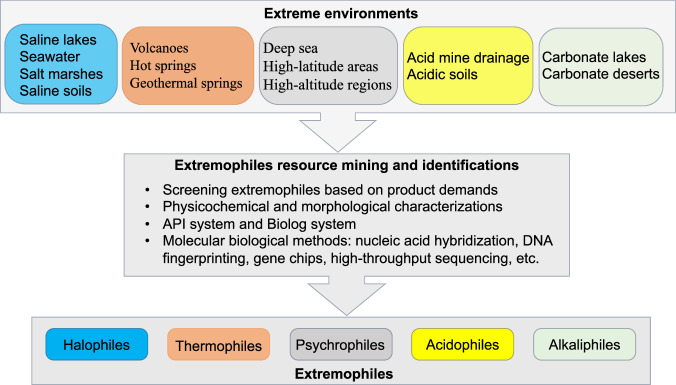
Extremophiles and resource mining. The Earth offers various environments as habitats for the growth of extreme microorganisms, providing us with extreme microbial resources. Multiple methods are being developed to facilitate extremophile-resource mining.

Extremophiles demonstrate remarkable diversity and are mainly classified into halophiles, thermophiles, psychrophiles, acidophiles, alkaliphiles, and xerophiles based on their ability to inhabit and thrive under specific conditions, ranging respectively from high salinity and high or low temperature to acidic, alkaline or dry conditions (Fig. 1; Shu and Huang, 2022; Wang et al, 2024). The metabolic and enzymatic diversity of extremophiles provides them with a unique adaptability to harsh and unstable environmental conditions (Shu and Huang, 2022). These adaptability mechanisms hold great potential for industrial applications (Ye et al, 2023; Blombach et al, 2022).

The metabolic and enzymatic diversity of extremophiles provides them with a unique adaptability to harsh and unstable environmental conditions.

Halophiles inhabit hypersaline environments that are ubiquitous across diverse geographical regions on Earth, including saline lakes, salt pans, salt marshes, seawater and saline soils (Ye et al, 2023; Blombach et al, 2022; Chen and Jiang, 2018). They maintain their osmotic balance via K^+^/Na^+^ antiporter proteins enabling iso-osmolarity between the cell interior and exterior to prevent dehydration, or via the synthesis of compatible solutes especially ectoine. By way of example, *H. bluephagenesis*, a member of the halophiles, synthesizes ectoine to maintain its osmotic balance in seawater (Hu et al, 2024).

Thermophiles are mainly distributed in high-temperature areas including volcanic lakes, hot springs and geothermal springs. They have evolved mechanisms for heat tolerance based on the thermal adaptability of cell membranes with an increased ratio of saturated to unsaturated fatty acids, high G/C content of nucleic acids, thermostable proteins and overall temperature-tolerant metabolic activities (Shu and Huang, 2022). The Taq-DNA polymerase used for PCR, for instance, can tolerate temperatures exceeding 90 °C without losing its activity; it originates from the thermophilic bacterium *Thermus aquaticus* that lives in hot springs.

Psychrophiles are prevalent in cold regions such as the deep sea, high-latitude areas, and high-altitude regions and uniquely adapted to their environments via enhanced cell membrane fluidity, reduced protein misfolding at low temperatures, and cryoprotectant-related proteins (Shu and Huang, 2022). The cold-active enzymes produced by psychrophiles exhibit high catalytic activity even at low temperatures, holding much prospects for widespread applications in agriculture, food, cosmetics and detergent industries (Wang et al, 2024).

Acidophiles inhabit acidic environments such as acid mine drainage and acidic soils and adapt by utilizing unique transmembrane potential differences and chaperone-based stress-response systems to buffer acid stress, by restricting cell-membrane permeability to prevent proton influx into the cytoplasm, and by enhancing heavy-metal tolerance (Shu and Huang, 2022). Alkaliphiles, on the other hand, are mainly found in extreme alkaline environments like carbonate lakes and deserts and maintain intracellular pH homeostasis via energy-dependent proteins and proton transporters. Their preference for an alkaline environment enable the use of CO_2_ as a carbon source which has never been exploited so far. These extremophiles have widespread applications in papermaking, detergent enzymes, cyclodextrin production, cosmetics, food and alkaline wastewater treatment (Shu and Huang, 2022; Wang et al, 2024).

## Advantages of next-generation industrial biotechnology

The Earth, with its varied environments ranging from the equator to the poles, from meadows to mountains, and from deserts to oceans, offers a multitude of habitats populated by extreme microorganisms. Their presence and the exploitation of their unique genetic and metabolic resources is the prerequisite for Next-Generation Industrial Biotechnology, which has several advantages over ‘classical’ biotechnology (Chen and Jiang, 2018).

Indeed, traditional industrial biotechnology based on conventional bacterial or yeast chassis encounters challenges regarding its efficiency and scalability (Chen and Jiang, 2018). Key problems include a high susceptibility to microbial contamination and the necessity for complex and costly sterilization procedures, significant consumption of freshwater resources, and difficulties of scaling up production processes. Although the rapid development of molecular engineering has enabled efficient customization of microorganisms, it still remains challenging to modify inherent traits, such as improving their resistance to contamination or the ability to use seawater. Furthermore, fermentations using standard chassis are often discontinuous owing to the need for regular cleaning and sterilization, which results in operational inefficiencies and creates substantial barriers to scaling up production (Ye et al, 2023; Chen and Jiang, 2018). Such systems also suffer from unstable and uneven product quality, leading to variability in outcomes and frequently necessitating downstream purification processes, adding to overall production costs and complexity (Chen et al, 2022). Next-Generation Industrial Biotechnology based on extremophiles could provide innovative solutions to these problems (Chen and Jiang, 2018).

… traditional industrial biotechnology based on conventional bacterial or yeast chassis encounters challenges regarding its efficiency and scalability.

Extremophiles are adapted to thrive in harsh environments such as high salinity, elevated temperatures, and extreme pH conditions, which naturally prevent microbial contamination. As a result, extremophiles enable open and continuous fermentation, obviating the need for sterilization and complex environmental controls. For example, *Halomonas* spp. grows in high-salt and high-alkaline conditions where most other microorganisms cannot survive. This reduces the need for expensive equipment, notable stainless-steel sterilizable fermenters, and enables the use of more economical materials such as ceramics or plastics reactors. Moreover, obviating the need for regular sterilization decreases energy consumption and operating costs, thereby enhancing both efficiency and sustainability.

Extremophiles are adapted to thrive in harsh environments [..], which naturally prevent microbial contamination.

Extremophiles also excel in utilizing non-food substrates, which addresses a critical challenge in sustainable biomanufacturing: the reliance on substrates derived from food crops (Zambare et al, 2011). Some can digest inexpensive raw materials including agricultural residues, lignocellulosic biomass or even gas substrates to produce high-value bioproducts. For example, halophiles, such as *Halomonas*, are grown in seawater using low-cost substrates in open, non-sterile, and continuous fermentation systems to produce diverse biochemicals, including polyhydroxyalkanoates (PHAs), proteins, and extracellular metabolites (Chen et al, 2022). This approach avoids competition with traditional agricultural resources over arable land and freshwater. Such applications clearly demonstrate the great potential of extremophiles for sustainable and green biomanufacturing.

## Genetic and metabolic engineering of extremophilic workhorses

Synthetic-biology methods are becoming increasingly user-friendly and adaptable for application in non-model organisms, which helps to develop novel and unconventional microbial chassis for the biotech industry (Ye et al, 2023; Blombach et al, 2022; Chen and Jiang, 2018). The discovery of genetic elements including promoters, ribosome-binding sites, insulators to protect transgenes from genomic effects, terminators to release RNA from the transcriptional machinery and of course CRISPR/Cas, as well as the continuous enrichment of transcriptional regulatory factors provide the essential basic gene for circuit and metabolic pathway design in extremophiles for industrial applications.

Taking halophile *Halomonas bluephagenesis* as an example, we constructed a constitutive promoter P_*porin*_ library for regulating gene expression by which A T7-like inducible system enabled inducible gene expression (Chen et al, 2022). In addition, an orthogonal system with multiple inducible promoters has facilitated selective gene expression (Ma et al, 2023). Recently, a biosensor regulatory system has been developed based on the polyhydroxyalkanoates (PHA) granule-binding proteins PhaR-phaP regulatory mechanism to synchronize the changes of poly-3-hydroxybutyrate (PHB) with the strength of its promoter (P_PhaP1_). It allows PHA accumulation along with enlarged shapes via increasing expression of the morphogenesis factor MinCD for self-induced synthesis of PHB and inducible cell morphological extension without the need for additional inducers (Zheng et al, 2024). Moreover, gene-editing technology permits precise design and efficient modification of organisms at the genomic scale. For example, CRISPR/Cas9 enables the insertion or deletion of gene fragments into the genome of *H. bluephagenesis*, while CRISPR/AID creates base mutations and CRISPRi reduces transcription of target genes.

Various strategies are now employed to modify metabolic pathways in extremophilic cells to obtain high-performance engineered strains with enhanced metabolic flux of target products. Carbon-source utilization pathways and substrate spectra are expanded to reduce raw material costs. For example, the secretory system of halophile *H. bluephagenesis* was engineered for growth on starch to produce PHA and other chemicals more cost-effectively. Increasing cell permeability or weakening substrate efflux transporters were used to elevate intracellular substrate concentrations to improve bioproduction (Ye et al, 2023). Here, LysP acting as a specific lysine-uptake transporter was overexpressed in engineered *H. bluephagenesis* and the resulting improved lysine permeability enhanced the production of cadaverine, a precursor for various pharmaceuticals (Zhao et al, 2022). Cell morphology was engineered by overexpressing or knocking out genes encoding morphological proteins MinCD or MreB to enlarge cellular volume to contain more or larger PHA granules (Zheng et al 2024).

The integration of synthetic biology and computational tools will further enhance the versatility of extremophiles. Predictive modeling of metabolic pathways based on AI and high-throughput screening will improve the design of regulatory circuits and metabolic pathways to enhance bioproduct formation in the selected extremophile strains, all while more genetic elements for circuit designs in extremophiles will be uncovered to benefit biotech applications.

## Applications of extremophiles in biomanufacturing

The main purpose of biomanufacturing is the sustainable production of fine chemicals and raw materials for the chemical or pharmaceutical industry. With the rapid advancement of synthetic biology and gene-editing technologies, extremophiles have emerged as promising platforms for producing a wide array of valuable compounds while offering distinct advantages in terms of efficiency, stability, and environmental impact.

Indeed, the study and application of extremophiles have gained significant attention from the chemical industry in recent years. For instance, a thermophilic *Geobacillus* sp. Iso5 isolated from hot springs produces a highly heat-resistant α-amylase with optimal activity at 140 °C (Zambare et al, 2011). Such thermostable enzymes are invaluable in the biorefining industry, particularly in high-temperature processes such as bioethanol production. Operating at elevated temperatures not only enhances catalytic efficiency but also mitigates risks of contamination, reduces production costs, and streamlines operations. Thermophiles are also known for their ability to produce extracellular polysaccharides that exhibit remarkable heat resistance. These thermodynamically stable compounds hold significant potential in industries beyond biofuels. For example, their application in the formulation of heat-stable emulsions for cosmetics, such as beauty creams, presents a novel application with high demand. Halophiles grown in seawater have already demonstrated significant potential for manufacturing high-value products like biodegradable plastics and amino acid derivatives (Zheng et al, 2024; Zhao et al, 2022).

Halophiles grown in seawater have already demonstrated significant potential for manufacturing high-value products like biodegradable plastics and amino acid derivatives.

**Figure 2:**
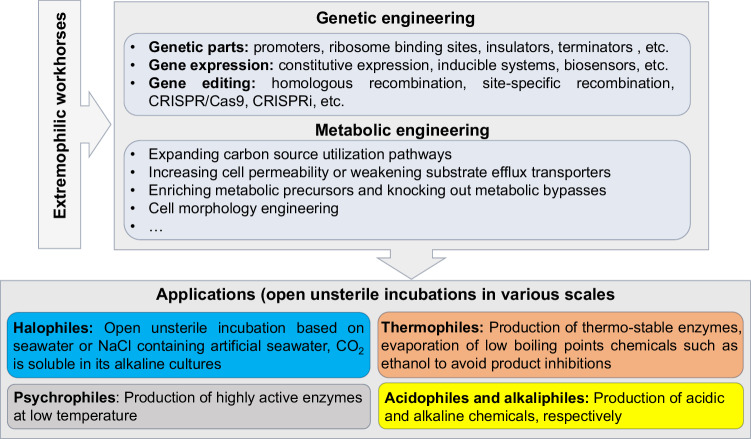
Engineering extremophiles and applications. Multiple tools and various strategies have been applied to the metabolic engineering of extremophiles. With the rapid advancement of synthetic biology and gene-editing technologies, extremophiles have emerged as promising platforms for producing a wide array of valuable enzymes, chemicals, and biomaterials.

The unique adaptations of extremophiles to harsh environments contribute to the production of novel bioactive compounds, which are typically absent in conventional microbial systems. By way of example, research investigated the diversity and bioactivity of actinobacteria isolated from Signy Island of Antarctica (Pan et al, 2013). The study identified numerous strains with the capacity to produce antimicrobial compounds, which could inspire the discovery and development of new antibiotics derived from extremophiles (Pan et al, 2013). The role of extremophile-derived metabolites in cancer therapy has also attracted growing interest (Thumar et al, 2010). For example, seven secondary metabolites were isolated from the halophilic bacterium *Nocardiopsis lucentensis* DSM 44048, including a benzoxazole derivative Nocarbenzoxazole G, which demonstrated selective cytotoxicity against HepG2 liver cancer cells and HeLa cervical cancer cells, highlighting its potential as a therapeutic agent (Thumar et al, 2010). Generally, the unique bioactive properties of extremophile-derived metabolites and enzymes present novel opportunities for innovation in biomedicine (Fig. 2).

Generally, the unique bioactive properties of extremophile-derived metabolites and enzymes present novel opportunities for innovation in biomedicine.

Engineered extremophiles to synthesize specific food ingredients or bioactive components broaden their applications in the food sector. For example, carotenoids are widely used as dietary antioxidants to prevent oxidative damage and protect human health. *Haloterrigena thermotolerans* K15, a halophilic and thermophilic archaeon isolated from Turkey, was metabolically engineered for carotenoid biosynthesis, paving the way for large-scale industrial production using extremophiles (Kesbiç and Gültepe, 2022). Another instance is gamma-aminobutyric acid (GABA), a non-protein amino acid with numerous biological functions including enhancing brain activity, calming the nervous system, improving lipid metabolism, and reducing blood pressure. It was coproduced by engineered *H. bluephagenesis* with PHA, yielding 357 g/L GABA together with PHA comprising 72% of the cell dry weight (Zhang et al, 2024). The co-production system not only maximizes resource utilization but also aligns with the principles of sustainable and efficient biomanufacturing.

Extremophiles also contribute to sustainable agriculture and improved productivity to address the resource limitations of traditional agriculture and the environmental challenges posed by livestock production. For example, 1,5-pentanediamide is a valuable C5 compound with diverse chemical and biological activities. It plays essential roles in plant physiological processes, including cell division and growth, development of pistils and stamens, regulation of senescence, and fruit maturation. These activities collectively enhance fruit development and increase crop yield. *H. bluephagenesis* was engineered to produce1,5-pentanediamide and achieved a high yield of 118 g/L (Zhao et al, 2022). In livestock production, the utilization of extremophile-derived enzymes as feed additives showed great promise in improving feed digestibility, conversion efficiency, and animal health. For example, the acid-resistant amylase produced by acidophile *Penicillium oxalicum* GZ-2 enhanced the nutritional value and utilization efficiency of animal feed, leading to better digestion, improved growth performance, higher productivity and reduced feed waste (Wang et al, 2024).

## Future perspectives

Due to the extreme environmental conditions of their specific habitats, extremophiles have evolved unique metabolic and enzymatic diversity. The rapid advancement of molecular engineering tools now allows for the efficient customization of these microorganisms in terms of genetic and metabolic engineering. Moreover, synthetic biology tools have become easier to adapt and apply to non-model microorganisms, facilitating the editing and modification of extremophile genomes for biomanufacturing. Currently, the field of biomanufacturing utilizing extremophiles encompasses a wide range of sectors, including chemicals, pharmaceuticals, food and agriculture, where they synthesize high-value compounds, with several achieving industrial-scale production yields. For instance, the industrial fermentation production of PHA utilizing *H. bluephagenesis* as a chassis has reached the kiloton scale, and a 10,000-ton production line is now under construction, scheduled to be completed in April this year.

However, compared to standard model microorganisms, the research endeavors to develop extremophile-based industrial chassis remain inadequate. Primarily, a majority of extremophiles still lack corresponding genetic engineering methods, which makes gene editing and metabolic engineering challenging. Furthermore, research on specific extremophiles is not as intensive as that on model organisms, resulting in insufficient understanding of these strains and slower progress. Moreover, the exploitation of extremophile resources continues to be both time-consuming and labor-intensive, particularly owing to the challenges associated with sampling in extreme environments.

… compared to standard model microorganisms, the research endeavors to develop extremophile-based industrial chassis remain inadequate.

To further propel the advancement of extremophile biomanufacturing, more research and efforts are needed, especially for highly acidic or alkaline products, or products that generate high osmotic pressure, products that require high activities under high or low temperatures, all of which are challenging to synthesize by classic microbial chassis. Aligning with the needs of synthetic biology and bioeconomy development, it is important to concentrate on key sectors to ensure that the research and efforts are targeted and effective, especially in hot or cold areas in the South or North, where thermophiles or psychrophiles can be grown normally without energy-intensive temperature regulation. Secondly, scientific and technological empowerment, coupled with innovation, is vital, as experiences with extremophiles are new to the global fermentation industry. Lastly, creating a conducive scientific, social and economic environment necessitates multifaceted approaches as more products will be produced by biotech industries along with CO_2_ reduction and energy saving as well as reduced process complexity. A rational and targeted approach to exploit the diversity of extremophiles will ensure that the fruits of research are transformed into practical applications that benefit industry and the environment.

## Supplementary information

Peer Review File
