# Supplementary material for: Next-generation biotechnology inspired by extremes: The potential of extremophile organisms for synthetic biology and for more efficient and sustainable biotechnology
Source: EMBO Rep. 2025 Feb 27;26(5):1191–5. doi: 10.1038/s44319-025-00389-6 (PMC11893800; doi:10.1038/s44319-025-00389-6)
Supplement: Supplementary file 1 — Peer Review File [file 44319_2025_389_MOESM1_ESM.pdf]

EMBO reports

Next-generation biotechnology inspired by extremes

Shuang Zheng, Mingwei Shao, Wanze Wang, and Guo-Qiang Chen

Corresponding author(s): Guo-Qiang Chen (chengq@mail.tsinghua.edu.cn)

Review Timeline:

Submission Date:	23rd Jan 25
Accepted:	3rd Feb 25

Editor: Holger Breithaupt

Transaction Report: This article was editorially reviewed.
